# Guidance and Standards for Breast Cancer Care in Europe

**DOI:** 10.1007/s13224-020-01316-6

**Published:** 2020-08-11

**Authors:** Rudy Leon De Wilde, Rajesh Devassy, Luz Angela Torres-de la Roche, Harald Krentel, Vlad Tica, Cristina Cezar

**Affiliations:** 1 University Hospital for Gynecology, Pius Hospital, University Medicine Oldenburg, Carl von Ossietzky University, Georgstrasse 12, 26121 Oldenburg, Germany; 2 Obstetrician and Gynecologist, Advanced Gynecological Minimal – Access Surgery, Dubai London Clinic and Speciality Hospital, Dubai, United Arab Emirates; 3 Clinic of Gynecology, Obstetrics, Oncology and Senology, Bethesda Hospital, Duisburg, 47053 Germany; 4 Obstetrics and Gynecology Department, University Regional Hospital, Ovidius University, Constanta, Romania

**Keywords:** Breast cancer, Clinical practice guidelines, European countries, Discrepancies

## Abstract

The increasing incidence and mortality rates of breast cancer have led to the necessity of initiating and developing clinical practice guidelines in order to optimize cancer control and provide patients with the best care. These guidelines are either national or issued by reputed relevant European societies—like European Society for Medical Oncology. Many of the recommendations are concordant in-between the guidelines. However, there are still considerable discrepancies to be noted between guidelines from different European countries, which could hinder physicians from implementing their recommendations. The present paper summarizes and compares the recommendations included in the various European guidelines.

## Introduction and Method

According to the European Society for Medical Oncology (ESMO), the incidence of breast cancer (BC) estimated in 40 European countries in 2012 was about 94.2/100,000 women, with a mortality rate of 23.1/100,000 [1]. Options for the treatment of BC include surgery, radiotherapy, chemotherapy, anti-HER2 therapy, and endocrine therapy, either sequentially or concomitantly. Although treatment success and prognosis are influenced by intrinsic tumoral factors, drug side effects, inherent toxicities of therapies, and therapy adherence. In order to optimize patient care, clinical practice guidelines (CPG) are formulated by committees of experts as statements that include evidence-based protocols and recommendations for specific patient profiles [2–5]. CPG recommendations also emphasize the importance of disease diagnosis and treatment being managed by an expert interdisciplinary team who base their treatment approach on the initial spread of the disease, the patient’s clinical condition and autonomy, as well as the availability of resources. However, few comparisons between these guidelines exist in the literature.

Here, we present a restricted comparison between several European guidelines regarding invasive early BC, according to their latest available versions: ESMO, DKG German Cancer Society—Interdisciplinary S3 guidelines for the diagnosis, treatment and follow-up care of BC [4], UK - NICE (National Institute of Clinical Excellence)—Early and locally advanced breast cancer: diagnosis and treatment [6], UK - SIGN (Scottish Intercollegiate Guidelines Network)—Management of breast cancer in women [7], SCR Swedish cancer register [8], the French Breast Cancer Intergroup [9, 10] and the Romanian Breast Cancer Clinical Guideline [11]. Whenever it was possible, we included the level of evidence reported by the cited resource.

## Breast Cancer Screening

According to ESMO guidelines, 18 countries from the EU offer mammography screening programs for the early detection of BC [1]. Screening programs, which consists of offering mammograms to women aged between 50 and 70 years, have been proven to reduce BC-related mortality [12]. ESMO recommends periodically mammography screening for women between 50 and 69 years old (IA) [1]. More specific are the NICE and German guidelines which recommend mammography for women between 50 and 70 years old every 3 and 2 years, respectively [4, 6]. ESMO also recommends yearly screening with MRI and mammography together or alternating every 6 months for women with a family history of BC, starting 10 years prior to the diagnosis age of the earliest case in the family (IIIB) [1].

EBCOG (European Board and College of Obstetrics and Gynaecology) Standards of Care stress the importance of women’s accessibility to “established and validated methods of screening”, in well-established, well-equipped and well-staffed screening units, based on written protocols [13].

The ESMO guidelines and EBCOG Standards of Care advise considering the possibility of false positive and false negative results of BC screening and recommend informing the patient regarding these issues [1, 13].

## Breast Cancer Diagnosis

NICE and ESMO guidelines recommend that patients with BC should be referred to specialized units [1, 13], with written protocols for diagnosis and management of BC [13]. These units must offer access to a multidisciplinary team [14] including personnel specialized in BC care: surgeon, radiation oncologist, medical oncologist, radiologist, pathologist, breast nurse [1] (IVA) and psychologist [13].

All guidelines recommend that the clinically suspected diagnosis must be associated with imaging (mammography, breast ultrasound or MRI) and confirmed pathologically. The imaging diagnosis of BC includes bilateral mammography and ultrasound of breast and regional lymph nodes. NICE guideline recommends a triple assessment (clinical assessment, mammography and/or ultrasound and biopsy) at the first visit to the breast clinic [6]. None of the guidelines recommend routine MRI. The ESMO guidelines recommend MRI in case of familial cases of BC with BRCA mutations, breast implants, multifocal BC (IIIB), to assess the response to chemotherapy, or in the case of inconclusive evidence (IIIA) [1]. Both the ESMO and the NICE guidelines recommend an MRI if the clinical examination and imaging is inadequate, in case of lobular BC (IIIB). NICE guidelines further recommend MRI in case of breast density that hinders the ultrasound examination [6].

ESMO guidelines recommend that the pathological confirmation be established by core needle biopsy, preferably obtained by ultrasound or stereotactic guidance. Core needle biopsy is mandatory in the case of planned preoperative systemic therapy in order to provide the diagnosis of invasive disease and the biomarkers status (IIIA). The pathological report should offer information about the histological type and grade of tumour, hormonal status (ER, PR), HER2 neu and Ki 67 status (IIIA) [1], an aspect also supported by NICE guidelines 2018 [6]. During the biopsy, it is recommended to place a marker (clip) to assure the correct surgical removal of the affected area (VA) [1]. NICE guidelines recommend confirmation by either core biopsy and/or fine needle aspiration cytology [6]. Romanian guidelines recommend the diagnosis of BC to be confirmed either by fine needle cytology, or by open (surgical) biopsy or Tru-cut needle [11].

## Breast Cancer Staging

According to ESMO [1] and NICE guidelines [6], the lymph nodes should be evaluated preoperatively by clinical examination and ultrasound (IIIA). All guidelines recommend cytological/histological examination of suspicious axillary lymph nodes. Given the fact that asymptomatic metastases in early BC are rare, ESMO guidelines recommend that staging be directed to locoregional disease in asymptomatic patients (IIID) [1]. Additional radiological investigations such as CT and bone scan are indicated in case of large tumours (> 5 cm), positive axillary nodes, or histological aggressive tumours (IIIB). A PET/CT examination may be useful if the results from conventional methods remain ambiguous (VA). Furthermore, PET/CT is indicated in high-risk patients, who would benefit from neoadjuvant chemotherapy, as well as for locally advanced BC or inflammatory disease, with an increased risk of metastasis (VB). Sonographic cardiac evaluation is necessary for patients who benefit from (neo) adjuvant chemotherapy with anthracyclines and/or trastuzumab (IA) [1]. Romanian guidelines also recommend further investigation (bone X-rays, bone scintigraphy, computed tomography scans of thorax, abdomen, head) only in the case of symptomatic patients (IIIB) [11].

## Breast Cancer Treatment

The comparison is limited to the following treatment modalities for BC:

- Surgery: breast conserving therapy, mastectomy, sentinel-node biopsy and axillary dissection
- Radiotherapy following surgical methods, either breast conserving or mastectomy
- Chemotherapy and or anti-HER2 therapy
- Endocrine therapy in pre- and postmenopausal patients

The therapeutic strategy depends on many factors like tumour biology, localization and tumour expansion (size and location of primary tumour, multifocality/multicentricity, the number and extent of lymph nodes), as well as individual considerations such as age, general health status and individual preference [1].

### Surgical Treatment

The breast conserving surgery (BCS) [15] is recommended by all guidelines if clear resection margins can be achieved and if the ratio between tumour size and breast volume is appropriate [1, 4, 6, 11]. Regarding the clear margins of resections, an explicit distance of 1 mm from all sides of the tumour is recommended by DKG [4]. NICE guidelines recommend a minimum of 2 mm radial margin of excision for conserving surgery for DCIS [6]. Furthermore, according to the NICE guidelines as of July 2018, if invasive cancer/DCIS is found within the 2 mm limit, an individual approach should be taken into consideration and the benefits and risks of further surgery should be discussed individually with the patient [6]. Both NICE and ESMO guidelines recommend offering immediate reconstructive surgery postmastectomy, the use of silicone gel implants being a safe and acceptable method of choice (IIIA) [1, 16]. Oncoplastic BCS should be taken into consideration, in order to improve the aesthetic outcomes, especially in women with large breasts [17].

The CPGs are generally concordant in respect to the necessity of axillary staging and SLNB (sentinel lymph node biopsy) [4]. According to ESMO, the SLNB has replaced routine axillary dissection as a standard of care in early BC, for patients with negative node—disease (IIA) [18]. ESMO guidelines state that no further axillary procedures are required in patients with limited SLNB involvement or presence of isolated tumour cells (< 0,2 mm) in the SLN, who benefit from breast irradiation (IIB) [1]. This assertion is backed-up by other European guidance [6] or meta-analysis [19]. According to NICE 2018, no further axillary dissection is required in the BCS patients with 1 or 2 sentinel lymph node macro-metastases, planned for whole-breast radiation and systemic, including endocrine therapy [6].

French guidelines propose axillary dissection in case of micro-metastasis without systemic treatment, as a significant deviation from other international recommendations [9]. This discordance was also noted in a recent French review of national and international guidelines for SLNB and complementary axillary dissection in BC. The authors conclude that the guidelines cannot be applied to all clinical cases, but it is necessary to undertake an individual decision by a multidisciplinary team [10].

Romanian guidelines recommend that, when choosing conservative surgery, level I and II lymphadenectomy should also be performed [11].

### Radiotherapy

Radiotherapy is necessary after BCS [4], and it is recommended by all guidelines. According to ESMO, postoperative radiotherapy represents a substantial part of the multidisciplinary treatment of early BC, plays an important role in the control of local disease, enables the BCS and improves the patients’ survival rates (IA) [20].

NICE guidelines 2018 recommend whole-breast radiotherapy in patients with invasive BC treated with BCS with clear margins and recommend partial breast radiotherapy for those patients with a low risk of recurrence or with an indication of endocrine therapy for ≥ 5 years. They also recommend considering not implementing radiotherapy for patients with invasive BC treated with BCS, with clear margins who have a very low absolute risk of recurrence and who are willing to have endocrine therapy for ≥ 5 years [6]. However, randomized studies on requirement of additional dose of radiotherapy for tumour bed are lacking [1, 21]. The main differences in guidelines' indications for postmastectomy radiation are summarized in Table 1.

**Table 1:** Main differences in guidelines´ indications for postmastectomy radiation

Guideline (Refs.)	Radiotherapy is recommended
SIGN 2013 [7]	Radiation recommended in all cases
ESMO 2015 [1, 22]	High-risk patients with ≥ 4 positive lymph nodes and/or T3-T4 tumours (IA)
SCR 2015 [8]	pN > 3
DKG 2018 [4]	pTt4
	pT3pn0r0 in case of L1, G3, premenopausal, age < 50 years old
	R1/R2 resection
	Pn + (> 3)
NICE 2018 [6]	In the case of relapse (pN > 3, positive margins)
Romanian guidelines [11, 21]	Positive margins or margins < 1 mm, T3-T4 tumours, more than 3 positive axillar lymph nodes, positive axillar lymph nodes with capsular refraction
	The radiotherapy (boost) in DCIS may be considered in patients at higher risk of recurrence (IIIB)

### Chemotherapy

In relation to recommendations for chemotherapy, SIGN 2013 recommends it for most cases, but data regarding additional survival benefits of taxanes over anthracycline regimens are insufficient [7]. The DKG 2018 recommends chemotherapy for patients with high risk of recurrence. An adjuvant therapy with taxanes should be taken in case of positive axillary lymph nodes [4]. Romanian guidelines recommend chemotherapy in case of positive axillar lymph nodes (B), or (may be considered) in case of patients with a high risk of recurrence (B) [11].

The NICE 2018 guidelines recommends the use of docetaxel as part of adjuvant chemotherapy in cases with positive axillar lymph nodes [6, 23]; the regimens do not include paclitaxel as part of adjuvant therapy for positive lymph nodes. They recommend adding a taxane to an anthracycline containing regimen because of the benefits of reduced side effects, dosing frequencies and increased chance of surviving [6]. These guidelines recommend neoadjuvant chemotherapy in patients with ER-negative invasive BC, HER-positive invasive BC and recommends considering it for ER-positive invasive BC as an option to reduce the tumour size [6]. Another recommendation is to consider the neoadjuvant endocrine therapy for ER-positive postmenopausal patients if there is no indication for chemotherapy [6]. NICE also advocates that all these aspects should be discussed individually with the patient [6].

#### HER2 Positive Breast Cancer

With respect to anti-HER2 therapy with trastuzumab, guidelines differ in its use, concurrently or sequentially [4], due to the cardiotoxic effects, which are mentioned in all guidelines.

- DKG 2018: recommends the use of trastuzumab concurrently with taxane or sequential to anthracycline/taxane regimens [4]
- NICE 2018: recommends adjuvant therapy with trastuzumab for patients with ≥ T1c, HER-positive invasive BC, every 3 weeks for 1 year, and considering it for patients with T1a/T1b, HER-positive invasive BC [6].

The systematic treatment recommendations according to ESMO clinical practice guidelines [1] are as following:

- Luminal A-like: endocrine therapy (ET) alone or with chemotherapy, in case of G3, T3 or ≥ 4 positive lymph nodes (IA)
- Luminal B-like (HER2-negative): ET and chemotherapy, but not concomitantly (IID)
- Luminal B-like (HER2-positive): chemotherapy and anti-HER2 (trastuzumab) and ET (IA). Only for selected cases, in which chemotherapy is contraindicated or refused by the patients, ET and trastuzumab may be considered as acceptable (VA)

In patients with intermediate risk of relapse (ER +/HER2-N0), the decision regarding chemotherapy may be taken by using uPA-PAI1 tumour markers as they have a level I evidence as strong prognostic factors (IA) [24].

Other clinical biomarkers to be used for uncertain indication of chemotherapy are Mamma Print, Oncotype DX, Prosigna and Endopredict (IVA) [1, 25].

- HER2-positive (non-luminal): chemotherapy and anti-HER2 (trastuzumab) (IA) [1]
- Triple-negative (ductal): chemotherapy (IA) [1]

### Endocrine Therapy

#### Premenopausal

All guidelines propose therapy with tamoxifen as the treatment of choice for a 5-year period in premenopausal women [4]. ESMO guideline recommends tamoxifen treatment for 5-10 years (IA) [1]. The indication for ovarian suppression is less clear [4]. In agreement with the St. Gallen Consensus Conference from 2017, ovarian function suppression should be taken into consideration in young patients (≤ 35 years) or in the case of ≥ 4 positive lymph nodes [26], as it has been demonstrated that ovarian suppression reduces the risk of cancer recurrence in high risk cancers [27]. ESMO guidelines also recommend a case-by-case approach for ovarian suppression [1].

NICE 2018 [6], DKG [4], SIGN [7] and Romanian guidelines [11] recommend considering ovarian suppression in women with ER-positive invasive BC.

#### Postmenopausal

A significant discrepancy between guidelines occurs in regard to the topic of endocrine therapy in postmenopausal women [4]. The DKG recommends aromatase inhibitors (AI) as a treatment of choice but cite NICE and SIGN which recommend tamoxifen for patients with general risk (SIGN) or lower risk (NICE) [4]. ESMO and Romanian guidelines recommend AI and tamoxifen [1, 11]. The St. Gallen Panel 2017 [26] noted that the treatment with AI, compared to tamoxifen, is better in reducing the recurrence risk and improving survival rates, but that tamoxifen still remains appropriate for sporadic cases [26]. In the case of positive lymph nodes, HER2 positive, higher grade, lobular histology and higher Ki 67, treatment with AI seems to be the treatment of choice [28, 29].

NICE 2018 recommends AI for postmenopausal patients with medium to high risk of recurrence, and tamoxifen for low risk patients [6]. NICE 2018 recommends offering extended AI therapy (more than 5 years) for ER-positive postmenopausal women at medium/high risk of recurrence, who were treated with tamoxifen for 2–5 years, and consider it for patients with a low risk of recurrence [6]. NICE 2018 also recommends tamoxifen treatment for more than 5 years for ER-positive pre- and postmenopausal women with invasive BC [6]. NICE advises offering endocrine therapy after conservative surgery for ER-positive patients with DCIS if radiotherapy was recommended, but not administered and its consideration if radiotherapy was not recommended [6]. Furthermore, NICE recommends considering selective serotonin reuptake inhibitor antidepressants in patients with menopausal symptoms who are not taking tamoxifen [6].

## Level of Evidence and Grade of Recommendation

Regarding the level of evidence and grade of recommendation [30], some disparities between guidelines can also be noted. DKG uses the Oxford classification—10 categories of evidence level (1a to 5) and 4 categories of grade recommendation (A, B, 0, GCP) [31], NICE uses eight categories (1 ++ to 4) [6], and SIGN proposes the same NICE—eight categories (1 ++ to 4) and five grades of recommendation (A, B, C, D, E). The ESMO Clinical practice guidelines use 5 levels of evidence (I to V) and 5 grades of recommendation (A to E) [1]. Romanian guidelines use 4 categories of grades recommendation (A to E), and 6 levels of evidence (Ia, Ib, IIa, IIb, III, IV) [11].

## Follow-up and Survival of the Patients with BC

Cancer survival can be defined as the clinical period between primary curative cancer treatment and the time of death, while cancer survivorship refers to “a distinct phase in the cancer trajectory between primary treatment and cancer recurrence or end of life” [32]. The goals of follow-up are the early detection of local recurrence or contralateral BC, evaluation of possible treatment—related complications, as much as optimal support of patients to regain capacity to undertake their social and professional activities [1, 2]. However, the follow-up recommendations differ between guidelines.

ESMO guidelines state that in the first 2 years, medical visits at every 3–4 months are recommended; after 3–5 years, every 6 months; after this period, patients should be encouraged to visit annually [1]. Romanian guidelines recommend clinical examination every three months in the first year, every 6 months in the second year and yearly visits beginning with the third year [11].

Annual bilateral mammography is recommended by all guidelines (IIA) and ultrasound can be taken into consideration for lobular invasive carcinomas (IIIB)—cited here are ESMO’s levels of evidence/grades of recommendation [1]. NICE guideline does not recommend ipsilateral mammography after mastectomy [6].

ESMO recommends other factors to be considered during the follow-up period: lipid profile (VA), annual gynaecological ultrasound (VB) for patients receiving tamoxifen, regular bone density for patients under AI and diet counselling for obese patients (IIIB). Hormone replacement therapy is not recommended due to the increased risk of recurrence (IA) [1]. Romanian guidelines recommend routine complete blood count, alkaline phosphatase, and, also, yearly chest X-ray, abdominal ultrasound and gynaecologic examination [11].

EBCOG standards of care recommend patient centred care, with emphasis on the psychological aspects as well as the need to reduce patient anxiety and discomfort. To this end, physicians should receive regular training in communication skills and the delivering of bad news, as well as in the management of vulnerable patients [13].

Finally, the European NCCP (National Cancer Control Programmes) for BC are in the process of continuous innovation and development, their recommendations being updated annually or even more often based on evidence gathered from newly published, relevant clinical trials [33].

## Conclusion

Given the tendency of increased incidence and mortality rates due to BC, the existence of clinical guidelines has become of great importance in order to provide optimal BC treatment by identifying the risk factors, improving the screening methods, as well as intensifying the operative methods, application of adjuvant therapy and follow-up care. However, there are significant discrepancies within European guidelines, which could hinder physicians from implementing their recommendations. Therefore, reviewing and updating of the current guidelines is indispensable. Physician still have the responsibility to select the most appropriate management among the available alternatives in order to provide the best care for the patient dealing with this disease.
